# Characterization of the Spanish Pomegranate Germplasm Collection Maintained at the Agricultural Experiment Station of Elche to Identify Promising Breeding Materials

**DOI:** 10.3390/plants11091257

**Published:** 2022-05-06

**Authors:** Elena Zuriaga, Jitka Pintová, Julián Bartual, María Luisa Badenes

**Affiliations:** 1 Citriculture and Plant Production Center, Instituto Valenciano de Investigaciones Agrarias (IVIA), CV-315, Km. 10.7, 46113 Valencia, Spain; judypin86@gmail.com (J.P.); badenes_mlu@gva.es (M.L.B.); 2 Agricultural Experiment Station of Elche, CV-855, Km. 1, 03290 Alicante, Spain

**Keywords:** *Punica granatum*, microsatellite, diversity, structure

## Abstract

Pomegranates were one of the first domesticated fruit crops, and their long history resulted in the development of local cultivars all over the world. Spain is one of the main producers and exporters of this crop in the Mediterranean Basin, but in order to maintain the competitiveness of this crop, new varieties should be developed. For this purpose, the pomegranate germplasm collection hold at the Agricultural Experiment Station of Elche, a public institution dependent on the Valencian regional government, is an interesting tool. However, the detailed characterization of any germplasm collection is a fundamental requirement to be able to make the most of these resources, allowing to identify putative promising accessions and to optimize the design of the future crosses. In this work, the genetic diversity of 94 accessions of this collection was analyzed using 19 microsatellite markers. As a result, 85 different genotypes were identified. These genetic profiles could be useful for varietal identification. Despite this genetic diversity, no clear substructure was observed, except for the ornamental accessions, that could be related to the vegetative propagation of the species. Additionally, the morphological characterization of this collection has made it possible to identify some materials that may be of interest as a source of traits for breeding. Results presented here pave the way for further genetic analyses, allowing the selection of parents to obtain segregating populations, as well as their descendants by the use of molecular assisted selection.

## 1. Introduction

*Punica granatum* L., commonly known as pomegranate, belongs to the *Punica* genus, included in the order Myrtales, recently placed under the family Lythraceae [1]. Pomegranate, cultivated for more than 5000 years, was one of the first domesticated fruit crops [2]. Although it is currently grown in subtropical and tropical areas all over the world, pomegranates are native to Iran and surrounding regions, as suggested by de Candolle [3]. From there, it was introduced into North Africa and Europe through the Mediterranean basin, and also to the rest of Asia. Later, Spanish sailors and Jesuit missionaries introduced pomegranates into Mexico and California in the 1500s and it arrived to Florida 200 years later [4]. Pomegranates have been used for multiple purposes since ancient times, such as food for humans and animals, medicinal remedies, religious purposes or just as ornamental trees. Despite this, pomegranate is still kept as a minor horticultural tree crop.

The long history of pomegranate domestication resulted in development of local cultivars in the different areas where it spread [4]. Due to its propagation method, mainly by cuttings, main *P. granatum* cultivars found today reflect local priorities [4]. More than 500 cultivars have been named, probably including synonyms, but just 50 are widely cultivated [5]. Pomegranates have a high diversity in phenological and pomological traits, as observed in germplasm collections from Turkey, India, Pakistan and Europe [6,7,8,9,10]. A high diversity in their chemical profile, including relevant content in anthocyanin, sugars and organic acids, was also observed [11,12].

Several international efforts have been made in order to preserve pomegranate diversity (as revised by [13]). Remarkably, the Garrygala Research Station in Turkmenistan, the largest ex situ pomegranate germplasm collection in the world, holds 1117 accessions and the Vavilov Research Institute of Plant Industry in Russia also maintains 800 accessions. A more modest germplasm collection of ~225 accessions is kept at the Agricultural Experiment Station of Elche (EEA-Elx, Alicante, Spain), a public institution dependent on the Regional Government of Valencia. This collection includes wild relatives, landraces, cultivars, advanced breeder selections and hybrids. As Spain is one of the main producers and exporters of pomegranates in the Mediterranean Basin [14], this collection represents a very interesting resource especially for the development of new varieties adapted to this region. Among the different ecotypes or cultivated varieties, it is worth highlighting the importance of the sweet and soft-seeded ‘Mollar de Elche’ and ‘Valenciana’, the main cultivars-populations grown in Spain that differ mainly in the ripening period. In order to fulfil the market needs developing new varieties, a pomegranate breeding program started in 2008 at the EEA-Elx and the Valencian Institute of Agrarian Research (IVIA, Valencia, Spain).

As breeding objectives, pomegranates have been traditionally selected on the basis of fruit size, rind and aril colors, taste and juiciness, and yield level, although some desirable characteristics differed by region [4]. For instance, the traditional Indian and Spanish cultivars are characterized by soft seeds and low-acid taste, while in Israel some sweet-sour cultivars, such as Wonderful, are successful [3]. Modern breeding objectives are more ambitious. According to Holland and Bar-Ya’akov [4], new cultivars will combine traits related with productivity, fruit quality, physiological disorders, ripening time, pest management, postharvest, and health promoting ingredients. However, despite the efforts made so far, development of new genomic and biotechnological tools is still needed to achieve these goals. The identification of regions of the genome involved in the control of these traits is essential to increase the efficiency of breeding programs through the use of molecular markers for selection.

The detailed characterization of any germplasm collection is a fundamental requirement to be able to make the most of these resources, allowing to identify putative promising accessions and to optimize the design of the future crosses. Different kinds of molecular markers have been used for pomegranate genetic diversity studies. However, simple-sequence repeats (SSRs) and single nucleotide polymorphisms (SNPs) displaced other types of markers and are preferentially used nowadays. In our group we developed a collection of 117 microsatellite markers from the cultivar Mollar because they were scarce a decade ago [15]. Recent examples of genetic diversity analyses using SSRs are the ones conducted using germplasm from India [16,17], Pakistan [18], or the Slovenian and Croatian areas of Istria [19]. Regarding the use of SNPs, due to cost reduction of massive sequencing, the first attempt to study diversity at the genomic scale was carried out by Ophir et al. [20]. These authors selected 480 SNPs from an RNAseq experiment to screen the germplasm collection at Agricultural Research Organization (ARO) located at the Newe Ya’ar Research Center in northern Israel.

In this work, we analyze the genetic diversity present in 94 pomegranate accessions from the EEA-Elx/IVIA collection using 19 microsatellite markers. Moreover, some morphological traits have been analyzed in these accessions. This work is a new step to improve the characterization of our pomegranate germplasm collection, allowing to generate new tools to optimize its use, as well as to open a new line of work aimed at identifying genes of interest and to apply assisted selection in the breeding program.

## 2. Results

### 2.1. Germplasm Collection

Ninety-four accessions from the pomegranate germplasm collection maintained at the EEA-Elx (Alicante, Spain) were analyzed in this work (Table 1), as an effort to better understand the collection and to improve the efficiency of the ongoing breeding program. From them, 80 were imported from the National Clonal Germplasm Repository for Tree Fruit, Nut Crops, and Grapes in Davis (CA, USA), 1 from the Newe Ya’ar Research Center (Ramat Yishay, Israel) and 13 come from collection expeditions and crosses carried out by researchers from the EEA-Elx. Regarding the different geographical origins, accessions were grouped as 35 from Central Asia, 15 from North America, 13 from Southern Europe, 8 from Eastern Europe, 7 from the Middle East, 5 from Transcaucasia, 5 from East Asia, 1 from South Asia, and other 5 with unknown origin. In this work, two different accessions named as ‘Wonderful-1’ (No. 92) and ‘Wonderful-2’ (No. 93) have been included. Although both come from the Davis germplasm bank, ‘Wonderful-2’ was annotated as North American but ‘Wonderful-1’ has an unknown origin. Moreover, they showed differences for some pomological traits in our conditions.

**Figure 1:**
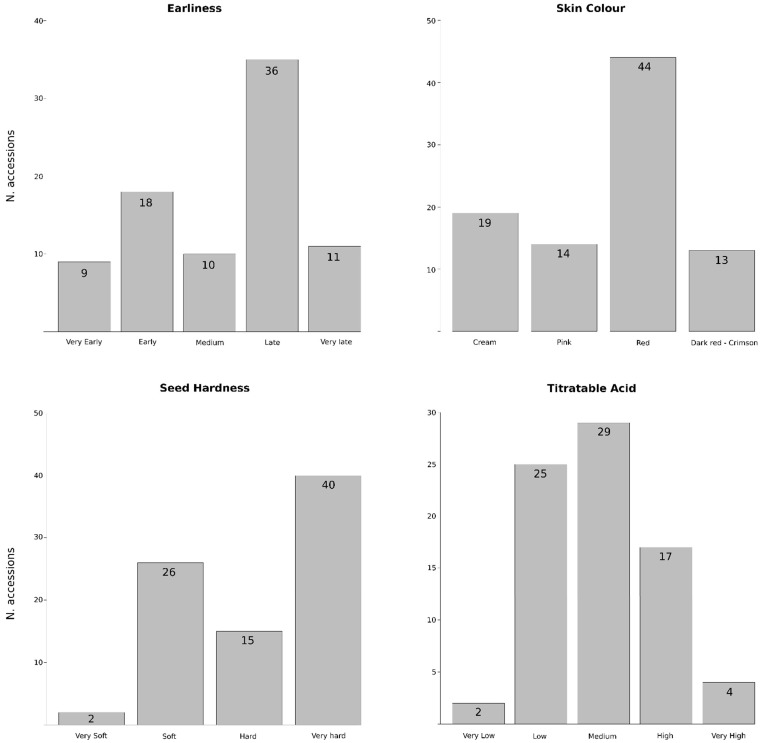
Phenotypic variation of the 94 pomegranate accessions for harvest period and three fruit quality traits (skin colour, seed hardness and titratable acid content). Number of accessions of each type is indicated.

Description of the morphological and pomological data of the analyzed accessions is summarized in Table S1. Some traits showed low variability, such as the ornamental use (with 9 of the 94 accessions classified as ornamental trees), the tree size (just 2 are dwarf and 1 semi-dwarf) or the fruit shape (the length/width ratio showed 2 accessions with moderately elongated fruits, 10 with elongated ones, and the rest with spherical to moderately compressed fruits). Regarding the sensitivity to the incidence of *Alternaria alternata*, a major pomegranate disease that impacts production worldwide, 15 accessions showed more sensitivity to fungal infection in stored fruits, due to the black heart detection within their fruits. On the other hand, higher variability was observed for other fruit traits (Figure 1). Regarding the harvest period or earliness, 9 and 18 accessions were classified as very early or early, respectively, becoming a good alternative to be used as donors in breeding programs. ‘Acco’ (No. 2) and ‘Wonderful-2’ (No. 93) have been considered as references for this trait, being classified in our conditions as early (end of August) and late (end of October), respectively. Fruits showed a wide array of rind colors ranging from cream or light yellow to dark red, although the red color is the most frequent (44 accessions). Soft-seeds, another desirable economic trait, are present in 28 accessions, including almost all the Spanish accessions. In fact, two of them, classified as very soft, are Spanish Mollar clones (‘Mollar-6’ (No. 60) and ‘Mollar-7’ (No. 61)). Finally, titratable acid (TA) content also displayed high variability, with 25, 29 and 17 accessions showing low, medium or high content, respectively. The two Spanish Mollar clones mentioned above showed very low TA content, while three accessions from Turkmenistan (No. 1, 54 and 59) and one from the former Soviet Union (No. 47) showed very high content.

### 2.2. Genetic Diversity, PIC and Cultivar Identification

The 19 SSRs, selected from [15], were polymorphic in the collection analyzed (Table S2). The number of different alleles detected varied from 3 to 13, with a mean number of 5.5. PIC values ranged from 0.178 (PGCT022) to 0.671 (PGCT087) (Table 2). Seventeen rare alleles, considered as polymorphic alleles having <1% frequency, have been identified (4 with PGCT110A, 3 with PGCT015, 2 with PGCT093B and PGCT111, and 1 in the case of PGCT038, PGCT083, PGCT088A, PGCT089, PGCT091 and PGCT093A). Interestingly, the accession from the former Sovier Union ‘Kaj Acik Anor’ (No. 46) and the Japanese ‘Nochi shibori’ (No. 65) showed three different rare alleles each one, while just one was detected in one accession from India (No. 27), Japan (No. 39) and Spain (No. 15), five from Turkmenistan (No. 57, 77, 79, 83 and 85) and three from USA (No. 55, 69 and 93). Observed heterozygosity ranged between 0.172 (PGCT022) to 0.554 (PGCT093A), while nonbiased expected heterozygosity [21] ranged between 0.179 (PGCT022) to 0.674 (PGCT087).

The 19 selected SSR markers allowed the identification of 85 different genotypes among the 94 studied accessions. A total of six groups of potential redundant accessions were identified from the 94 accessions as having identical SSR marker profiles within each group (Table 3). However, in some cases morphological or pomological differences were observed between them (Table S1). For instance, within group 2, ‘Kara bala miursal’ (No. 47), described as a bud sport of ‘Bala Miursal’ (No. 12), appeared with this one and also with ‘Crab’ (No. 20) and ‘Sakerdze’ (No. 74), and they showed some differences in earliness, skin color and also titratable acid content. However, the darkening of the skin was the only difference that we observed between ‘Cranberry’ (No. 21) and ‘Koinekasyrskii Kislosladkii Krasnyi’ (No. 51) accessions from group 3. A more detailed description of these accessions should be obtained in order to declare them as duplicates or synonymously mislabeled. In order to minimize bias, just one accession from each group was maintained for the subsequent analyses.

### 2.3. Population Structure and Genetic Relationships

**Figure 2:**
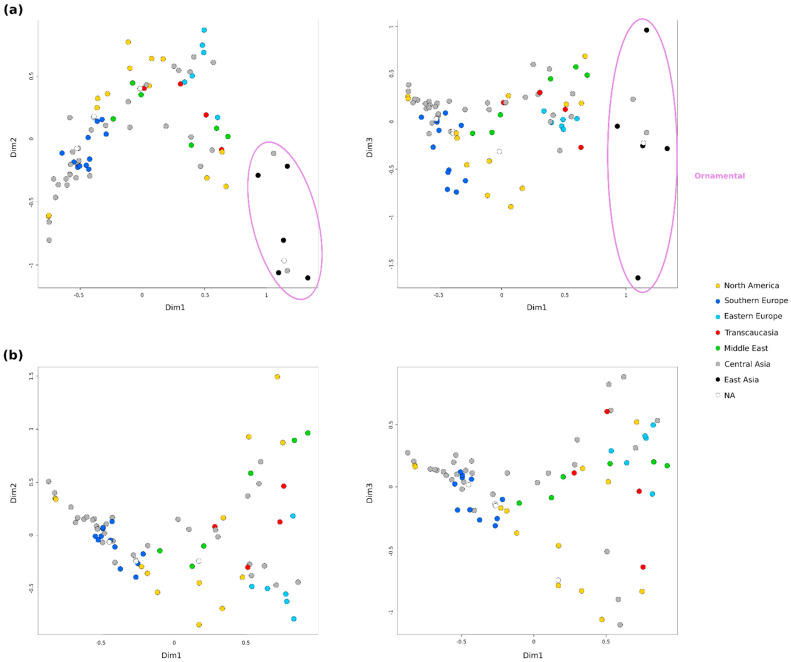
Factorial Correspondence Analysis (FCAs) of the accessions analyzed using 19 SSRs. Colors represent the putative region of origin of the genotype. (**a**) FCA without the Indian ‘Elx-13’ accession (No. 27); (**b**) FCA without ‘Elx-13’ and the 8 ornamental accessions.

Relationships between accessions and population structure were explored using different approaches. First, several Factorial Correspondence Analysis (FCAs) were conducted to explore the patterns of variation in the collection (Figure 2 and Figure S1). For clarity purposes, the coordinates of each accession in each FCA can be checked in Table S3. In general, some grouping according to the broad geographical regions was observed. The accessions from Central Asia, the main number, appeared distributed throughout the graphs. However, two groups were observed within the accessions from North America and also from the Middle East. A clear separation was also observed between samples from Southern and Eastern Europe. The FCA including all 85 accessions, after removing the nine potential redundancies, explained 26.7% of the variability by the first 3 dimensions and showed the ‘Elx-13’ accession (No. 27) clearly separated from the rest by the third dimension (Figure S1). This accession is a seedling from India, the only one in this analysis coming from South Asia. Accordingly, a new FCA was performed without this accession (Figure 2a). In this case, the accessions appear more distributed and the first 3 dimensions explained the 26.81% of the variability. Ornamental accessions appeared grouped and separated from the rest by the first dimension. This group of 8 accessions included the 5 from East Asia (all Japanese), 2 from Central Asia (No. 24 and 91) and 1 of unknown origin (No. 41). Close to them, in the central part of the first graph, appeared one group from North America (No. 19, 25 and 55) and another from the Middle East (No. 5, 23 and 87), jointly with some accessions from Central Asia (No. 42, 71, 77 and 94), Transcaucasia (No. 9 and 43) and Eastern Europe (No. 6). Finally, third dimension clearly separated the Japanese ornamentals ‘Ki-Zakuro’ (No. 50) and ‘Nochi shibori’ (No. 65), but also in a more subtle way other 5 accessions from North America (No. 7, 37, 38, 53, 69) and 5 from Southern Europe (No. 26, 29, 30, 31, 60).

Finally, a third FCA was performed without the 8 ornamental accessions in order to observe in more detail the relationships between the rest of the accessions (Figure 2b). In this case, the first 3 dimensions explained 27.49% of the variability. A greater dispersion of the accessions on the right half of the first graph was observed. In this case, the grouping described in the central position in the previous FCA was more clearly seen, but this time in the upper right part of the graph. Moreover, the other 5 accessions from Eastern Europe (No. 3, 8, 45, 73 and 76) appeared grouped with other 5 from Central Asia (No. 46, 49, 52, 79 and 84), one from North America (No. 53) and another one from Transcaucasia (No. 58). Contrary, almost half of the accessions from Central Asia appeared grouped with the ones from Southern Europe and a few from North America or the Middle East. A central position was occupied by a group of accessions from the Middle East (No. 2, 56 and 78), Central Asia (No. 35, 48, 59 and 71) and North America (No. 7, 21, 37, 38 and 69). Third dimension also separated 10 accessions, 5 from North America (No. 7, 37, 53, 55 and 69), 3 from Central Asia (No. 42, 77 and 94), 1 from Transcaucasia (No. 43) and 1 with an unknown origin (No. 16).

**Figure 3:**
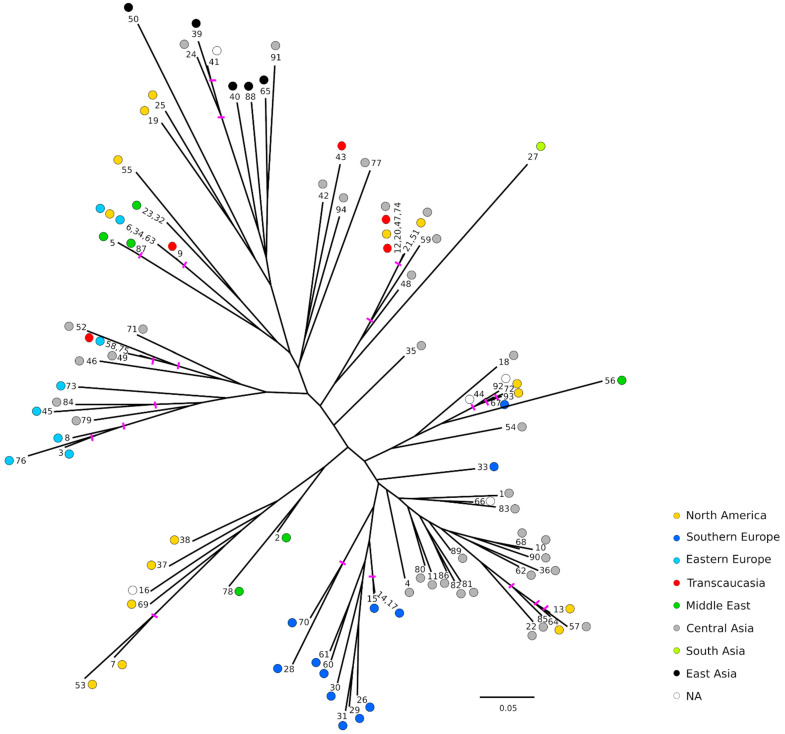
Neighbor-Joining tree using Bruvo’s distance [22]. Colors represent the geographical origin of the genotype. Pink bars indicate nodes with bootstrap support > 50. As just one accession belonging to the same redundant group has been used for the analysis, the rest of names have been included separated by commas.

As a second approach to explore the relationships between the accessions, a Neighbor-Joining tree was built using Bruvo’s distance [22] (Figure 3). Bootstrap supports were quite low overall, with values greater than 50 only on some outer nodes. In general, similar geographical grouping to those of the FCAs was observed. Ornamental accessions appeared also grouped, while Southern and Eastern European accessions appeared separated in two groups as observed before in the FCAs. It should be noted that among the Southern European accessions, all the Spanish ones appeared clearly grouped and close to a group of accessions from Central Asia. Similarly, the accessions from Eastern Europe appeared close to each other but in this case also intermingled with some materials from Central Asia. Regarding the accessions of unknown origin, in some cases it seems that their origins could be inferred. For instance, ‘Cana’ (No. 16) appeared clearly grouped with some accessions from North America, although according to the name it could be from the Middle East. Also, ‘Orange’ (No. 66) grouped with some from Central Asia, and ‘How Sweet It Is’ (No. 41) appeared grouped with the ornamental ones. Contrary, ‘Ink’ (No. 44) and ‘Wonderful-1’ (No. 92) appeared grouped with some accessions from different regions such as Central Asia (‘Chandyr’ (No. 18)), North America (‘Purple Heart’ (No. 72) and ‘Wonderful-2’ (No. 93)), Southern Europe (‘Palermo’ (No. 67)) and Middle East (‘Mahali Dezful’ (No. 56)).

Finally, a Bayesian-based population assignment allowing admixture was carried out using the Structure software [23] (Figure S2). Different methods have been suggested to select the value of the number of clusters (K) that best fits the data [23,24]. In this case, the best grouping number was 2 based on delta K method, but also the maximum likelihood of K was observed for K = 10 (Figure S2C,D). For this reason, the assignment of each accession to the different groups assuming K = 2 (Figure S2A) and 10 (Figure S2B) was inspected. However, a clear relationship was not observed either with the geographic distribution of the accessions nor with any of the morphological characters analyzed, not even if we focused only on accessions showing a membership coefficient Q > 0.90.

### 2.4. Genetic Differentiation of Geographical Groups

Taking into account that the classification in geographical regions used in this work reflects the putative origin of the samples, several diversity indexes were calculated in order to characterize these geographic groups (Table 4). Overall, Central Asia group was the most variable, but this could be biased due to the uneven sample sizes between groups. In order to minimize this bias, the normalized multilocus genotypes (eMLGs) can be used to compare the genotypic diversity among the populations. In this case, Central Asia and North American groups were the most diverse, followed by Southern Europe. Exclusive alleles were found in accessions from 5 groups. It is worth noting the high number of unique alleles identified in accessions from East and South Asia (6 and 5, respectively) despite the lower number of individuals analyzed from those regions, 5 and 1 respectively. The Simpson index, calculated as one minus the sum of squared genotype frequencies, showed the existence of great diversity in the collection, as values close to 1 imply that two randomly selected genotypes are different.

## 3. Discussion

Pomegranate was among the first fruit crops to be domesticated and has been cultivated for more than 5000 years [2]. This long history resulted in the development of pomegranate cultivars in the world that reflect the different tastes and priorities in each country [3]. Pomegranate production in Spain is mainly located in the Valencian Community (78.6%), basically in the Alicante province. This crop is very well adapted to this region as it is resistant to the hot dry climate and poor soils, tolerating calcareous soils with a degree of salinity [14,25]. Two pomegranate germplasm collections are maintained in the Alicante province. The first one is hold since 1992 by the Miguel Hernández University and maintains 59 accessions collected from different Spanish regions [9]. The second one is hold by the Agricultural Experiment Station of Elche/Instituto Valenciano de Investigaciones Agrarias (EEA-Elx/IVIA), belonging to the Valencian Regional Government, and currently maintains 225 accessions (including segregating populations) from 25 different countries.

In recent years, this crop has gained attention primarily due to its potential medicinal properties and its nutritional benefit in the human diet [26]. However, maintaining the competitiveness of this crop requires the development of new varieties that can meet the demands of consumers as well as the conditions imposed by climate change. The EEA-Elx/IVIA’s pomegranate germplasm collection is a valuable tool as a source of traits of interest for this purpose, but knowing its variability is what can make it useful. It is very important to characterize the collection, to know its strengths and weaknesses, identifying the genotypes that may be of interest for carrying out the crosses, as well as the need to incorporate other varieties that may have certain traits of interest that are not yet represented. This information has a direct interest for the pomegranate breeding program carried out at the EEA-Elx/IVIA but could also be useful for other breeding programs worldwide, by potentially identifying certain materials that may be of interest to them.

Main breeding goals to develop a new pomegranate cultivar are related with its destination for fresh consumption, industrialization or export. In general, an early ripening, attractive dark rind and arils color, presence of soft seeds and high antioxidant content are highly appreciated traits [14]. In this context, some promising materials have been identified in the collection, such as the 9 very early ripening accessions, the 13 with dark red rind color or the 2 accessions showing very soft seeds. These accessions could be good potential candidates as parents for crossing in the breeding program, but also for the generation of segregating populations that would allow us to undertake genetic studies. Moreover, phenotyping is important to identify possible environmental effects, despite being very costly in time and space. For instance, other authors suggested that rind and aril color can vary when grown in different regions [27]. In fact, some Spanish cultivars grown in Israel showed poorer and unattractive colors there than in Spain [3]. For this reason, it is essential to study the behavior of the materials in the regions where they are going to be grown.

Genotyping could also be useful to manage the germplasm collection as it could detect putative synonymies (identical accessions named differently) and homonymies (different accessions with the same name). This is a quite common problem in this species since the interchange of planting material must have been intense. Moreover, passport data in genebanks may not be as complete as desired, especially on the origin of the sample. For instance, in this work six small groups (with 2 to 6 accessions) with identical SSR profiles were identified. However, some morphological and pomological differences were observed within each group, so a more detailed description should be performed to confirm them as duplicates. These efforts will allow us to eliminate or at least reduce the redundancy within the collection.

Regarding the diversity observed, the microsatellites used in this work showed a great genotypic richness in terms of multilocus genotypes, so they are very informative in these collections. In this sense, 85 of the 94 accessions analyzed in this work showed a different genetic profile. The normalized multilocus genotypes eMLGs, that eliminates the influence of population size, showed similar values for Central Asia, North America and Southern Europe groups. Moreover, the accession from South Asia has shown a greater differentiation with respect to the rest of the materials under study. This is the seedling from India named ‘Elx-13’ (No. 27), that appeared separated from the rest of the analyzed accessions and showed 5 exclusive alleles, and has red rind color, soft seeds and low acidity in our conditions. Interestingly, all these traits are similar to those shown by ‘Mridula’ or ‘Bhagwa’ [3], Indian varieties extensively used for export to Europe. However, more work would be necessary to know if they are related or not.

Despite the genetic diversity observed in the EEA-Elx/IVIA collection, the materials do not show clear and differentiated groupings, except perhaps the varieties of ornamental use. This could be related to the vegetative propagation of the species, which in practice means avoiding frequent recombinations to obtain new generations through sexual reproduction. Similar results showing lack of significant genetic divergence by geographical origin were already observed by other authors. Genetic diversity of pomegranate germplasm from different origins has been previously analyzed using different morphological or pomological traits [9,12,28,29] and also different types of molecular markers as mentioned above. However, the comparison with the results from these works is complicated by the lack of concordance in the materials used in each case. In general, although some clusters are shown, the support for these classifications is often low. This may be indicating a relationship between the different materials that are not sufficiently separated.

In summary, results presented here pave the way for further genetic analyses, allowing the selection of parents to obtain segregating populations, as well as their descendants by the use of molecular assisted selection. Additionally, the phenotyping of new traits is in progress in order to increase the potential utility of the EEA-Elx/IVIA collection.

## 4. Materials and Methods

### 4.1. Plant Materials

Ninety-four pomegranate accessions from different origins were PCR screened in this work (Table 1). This collection is maintained at the Agricultural Experiment Station of Elche/Instituto Valenciano de Investigaciones Agrarias (EEA-Elx/IVIA), belonging to the Valencian regional government, and located in Elche, a city of the Alicante province, in southeastern Spain. Two leaf discs were collected from each accession, frozen in liquid N2 and stored at −80 °C before DNA isolation.

### 4.2. Morphological and Pomological Characterization

Eight interesting traits for pomegranate breeding were evaluated in this germplasm collection (Table 1). The traditional use of some varieties as ornamentals has been indicated. Tree vigour and some external fruit traits, such as the shape and rind color, were visually inspected. Regarding internal quality traits, seed hardness, titratable acid content (TA) and sensitivity to *Alternaria* in stored fruits were also screened. TA of fruit juice was determined by titrating 1 mL of juice sample with 0.1 mol/L sodium hydroxide to an end point of pH 8.1 and expressed as percentage of citric acid. TA values were classified as very low (<0.35 g/100 g), low (0.35–1.00 g/100 g), medium (1–1.50 g/100 g), high (1.50–2.0 g/100 g) and very high (>2.0 g/100 g). In order to detect the incidence of *Alternaria alternata* in stored fruits of these accessions, presence of symptoms consisting of internal black rot of arils and membranes was screened opening 100 fruits of each one [30]. Finally, the accessions were classified according to their fruit maturation period as very early (<20 August), early (20 August–20 September), medium (20 September–10 October), late (10–30 October) and very late (>30 October).

### 4.3. DNA Isolation and Microsatellite Analysis

DNA was extracted following the method described by Doyle and Doyle [31]. DNA quantification was performed by NanoDrop ND-1000 spectrophotometer (Thermo Fisher Scientific, Wilmington, DE, USA) and integrity was checked on 1% agarose gel.

Nineteen microsatellite (SSR) markers (Table 2) were selected according to their informative content from Soriano et al. [15]. SSR amplifications were performed in a final volumen of 20 µL containing 1× DreamTaq buffer, 0.2 mM of each dNTP, 20 ng of genomic DNA and 1 U of DreamTaq DNA polymerase (Thermo Fisher Scientific, Waltham, MA, USA) using a UNO96 thermal cycler (VWR, Radnor, PA, USA). Each reaction was performed in 20 µL with three primers: the specific forward primer of each microsatellite with an M13(−21) tail at its 5′ end (0.05 µM), the sequence-specific reverse primer (0.25 µM) and the universal fluorescent-labeled M13(−21) primer (0.2 µM) [32]. The PCR temperature cycling conditions were as follows: 94 °C for 2 min, then 35 cycles of 94 °C for 30 s, the optimized annealing temperature (Table 2) for 60 s and 72 °C for 90 s, finishing with 72 °C for 10 min. Allele lengths were determined using an ABI Prism 3130 Genetic Analyzer with the aid of GeneMapper software, version 4.0 (Applied Biosystems, Waltham, MA, USA).

### 4.4. Data Analysis

Histograms, obtained using R programming language, were used to visualize the distribution of the morphological and pomological traits in the collection.

For each microsatellite the number of alleles, their size range and polymorphism information content (PIC) were calculated. PIC was calculated based on allele frequencies of all cultivars analyzed as: PICi = 1 − Σpij^2^, where pij is the frequency of the jth allele for the ith marker locus and summation extends over n alleles. Observed (Ho) and expected (He [21]) heterozygosity were calculated using the Genetix program [33].

In order to determine the relationship of the accessions used, several factorial correspondence analyses (FCA) were carried out using the Genetix program [33]. Genetic distances between pairs of accessions were calculated using the Bruvo’s distance [22] and used to construct an unrooted neighbor-joining (NJ) phylogenetic tree The stability of the nodes was checked with 1000 bootstrap replicates. These analyses were conducted through the R package Poppr [34]. HyperTree software [35] was used to visualize the obtained trees. The number of multilocus genotypes found in each population (MLG), the expected number of MLG at the lowest common sample size (eMLG), the mean number alleles per locus (A), athe observed heterozygosity (Hobs) and the unbiased estimated heterozygosity (Nei’s gene diversity) (Hexp) were also calculated using the with R package Poppr [34].

The accessions were classified into genetic clusters using the Bayesian model-based clustering proposed by Pritchard and collaborators [23] implemented in STRUCTURE 2.3.4 (https://web.stanford.edu/group/pritchardlab/structure.html (accessed on 13 April 2022)). We used the basic admixture model with unlinked loci, correlated allele frequencies among groups and no prior population information. Twenty runs were performed for each number of populations (K) set from 1 to 13, with a burning period of 100,000, and a post-burning simulation length of 1,500,000 for each run. The most probable K-value was determined by Structure Harvester [36], using the log probability of the data [LnP(D)] and delta K (1 K) based on the rate of change in [LnP(D)] between successive K-values. For the optimal K-value, membership coefficient matrices of 20 replicates from STRUCTURE were used in CLUMPP [37] to generate an individual Q matrix. STRUCTURE PLOT webpage (http://omicsspeaks.com/strplot2/ (accessed on 13 April 2022)) was used to draw the STRUCTURE bar plots [38].

## 5. Conclusions

The present study provides a perspective of the genetic variation of the Spanish pomegranate germplasm collection maintained at the Agricultural Experiment Station of Elche/Instituto Valenciano de Investigaciones Agrarias (EEA-Elx/IVIA). Some promising materials with interesting morphological and pomological characteristics for breeding have been identified in the collection. Moreover, the SSR genotyping data provided valuable information for an effective management of the collection and the identification of the materials, as an interesting tool to increase the protection of breeder’s intellectual rights. Results presented pave the way for further genetic analyses that could increase the efficiency of the pomegranate breeding programs.

## Figures and Tables

**Table 1:** Accessions analyzed in this work.

Acc	Name	Region	Country	Original Germplasm Bank
1	15/4 Pamyati Rozanova	Central Asia	Turkmenistan	Davis, CA, USA
2	Acco	Middle East	Israel	Newe Ya’ar Research Center, Israel
3	Afganski	Eastern Europe	former Soviet Union	Davis, CA, USA
4	Agat	Central Asia	Turkmenistan	Davis, CA, USA
5	Alk Pust Ghermez Saveh	Middle East	Iran	Davis, CA, USA
6	Al-sirin-nar	Eastern Europe	former Soviet Union	Davis, CA, USA
7	Ambrosia	North America	United States	Davis, CA, USA
8	Apseronski	Eastern Europe	former Soviet Union	Davis, CA, USA
9	Apseronski krasnyj	Transcaucasia	former Soviet Union	Davis, CA, USA
10	Ariana	Central Asia	Turkmenistan	Davis, CA, USA
11	Azadi	Central Asia	Turkmenistan	Davis, CA, USA
12	Bala Miursal	Transcaucasia	former Soviet Union	Davis, CA, USA
13	Balegal	North America	USA	Davis, CA, USA
14	Borde-1	Southern Europe	Spain	EEA d’Elx, Spain
15	Borde-2	Southern Europe	Spain	EEA d’Elx, Spain
16	Cana	Unknown	Unknown	Davis, CA, USA
17	Casta del Reino	Southern Europe	Spain	EEA d’Elx, Spain
18	Chandyr	Central Asia	Turkmenistan	Davis, CA, USA
19	Chico	North America	United States	Davis, CA, USA
20	Crab	North America	United States	Davis, CA, USA
21	Cranberry	North America	United States	Davis, CA, USA
22	Desertnyi	Central Asia	Turkmenistan	Davis, CA, USA
23	Dorosht 5 hahanshahi Khoramabad	Middle East	Iran	Davis, CA, USA
24	Dotch Legrelley	Central Asia	Turkmenistan	Davis, CA, USA
25	Elf	North America	United States	Davis, CA, USA
26	Elx-11	Southern Europe	Spain	EEA d’Elx, Spain
27	Elx-13	South Asia	India	EEA d’Elx, Spain
28	Elx-6	Southern Europe	Spain	EEA d’Elx, Spain
29	Elx-7	Southern Europe	Spain	EEA d’Elx, Spain
30	Elx-8	Southern Europe	Spain	EEA d’Elx, Spain
31	Elx-9	Southern Europe	Spain	EEA d’Elx, Spain
32	Entek habi saveh	Middle East	Iran	Davis, CA, USA
33	Ermioni	Southern Europe	Greece	EEA d’Elx, Spain
34	Eve	North America	United States	Davis, CA, USA
35	Girkanets	Central Asia	Turkmenistan	Davis, CA, USA
36	Gissarskii Rozovyi	Central Asia	Turkmenistan	Davis, CA, USA
37	Golden Globe	North America	United States	Davis, CA, USA
38	Green Globe	North America	United States	Davis, CA, USA
39	Haku Batan	East Asia	Japan	Davis, CA, USA
40	Haku Taka	East Asia	Japan	Davis, CA, USA
41	How Sweet It Is	Unknown	Unknown	Davis, CA, USA
42	Hyrdanar x Goulosha	Central Asia	Turkmenistan	Davis, CA, USA
43	Hyrdanar x Kirmizy- Akbuh	Transcaucasia	former Soviet Union	Davis, CA, USA
44	Ink	Unknown	Unknown	Davis, CA, USA
45	Kaim Anor	Eastern Europe	former Soviet Union	Davis, CA, USA
46	Kaj Acik Anor	Central Asia	former Soviet Union	Davis, CA, USA
47	Kara bala miursal	Transcaucasia	former Soviet Union	Davis, CA, USA
48	Kara-Kalinskii	Central Asia	Turkmenistan	Davis, CA, USA
49	Kazake	Central Asia	former Soviet Union	Davis, CA, USA
50	Ki-Zakuro	East Asia	Japan	Davis, CA, USA
51	Koinekasyrskii Kislosladkii Krasnyi	Central Asia	Turkmenistan	Davis, CA, USA
52	Kunduzski	Central & South Asia	Afghanistan	Davis, CA, USA
53	Loffani	North America	United States	Davis, CA, USA
54	Machtumkuli	Central Asia	Turkmenistan	Davis, CA, USA
55	Mae	North America	United States	Davis, CA, USA
56	Mahali Dezful	Middle East	Iran	Davis, CA, USA
57	Medovyi Vahsha	Central Asia	Turkmenistan	Davis, CA, USA
58	Mejhos 6269	Transcaucasia	former Soviet Union	Davis, CA, USA
59	Molla Nepes	Central Asia	Turkmenistan	Davis, CA, USA
60	Mollar-6	Southern Europe	Spain	EEA d’Elx, Spain
61	Mollar-7	Southern Europe	Spain	EEA d’Elx, Spain
62	Myatadzhy	Central Asia	Turkmenistan	Davis, CA, USA
63	Nikitski ranni	Eastern Europe	former Soviet Union	Davis, CA, USA
64	No name	North America	United States	Davis, CA, USA
65	Nochi shibori	East Asia	Japan	Davis, CA, USA
66	Orange	Unknown	Unknown	Davis, CA, USA
67	Palermo	Southern Europe	Italy	Davis, CA, USA
68	Parfyanka	Central Asia	Turkmenistan	Davis, CA, USA
69	Phoenicia	North America	United States	Davis, CA, USA
70	Piñon tierno Blanca	Southern Europe	Spain	EEA d’Elx, Spain
71	Podarok	Central Asia	Turkmenistan	Davis, CA, USA
72	Purple Heart	North America	United States	Davis, CA, USA
73	Saartuzski	Eastern Europe	former Soviet Union	Davis, CA, USA
74	Sakerdze	Central Asia	former Soviet Union	Davis, CA, USA
75	Salavatski	Eastern Europe	former Soviet Union	Davis, CA, USA
76	Sejanec 2–5/8	Eastern Europe	former Soviet Union	Davis, CA, USA
77	Shainakskii	Central Asia	Turkmenistan	Davis, CA, USA
78	Shirin Pust Ghermez Saveh	Middle East	Iran	Davis, CA, USA
79	Shirin Zigar	Central Asia	Turkmenistan	Davis, CA, USA
80	Sirenevyi	Central Asia	Turkmenistan	Davis, CA, USA
81	Sogdiana	Central Asia	Turkmenistan	Davis, CA, USA
82	Sumbar	Central Asia	Turkmenistan	Davis, CA, USA
83	Sumbarskii	Central Asia	Turkmenistan	Davis, CA, USA
84	Surh-anor	Central Asia	former Soviet Union	Davis, CA, USA
85	Sverkhranniy	Central Asia	Turkmenistan	Davis, CA, USA
86	Syunt	Central Asia	Turkmenistan	Davis, CA, USA
87	Tabestani Males Biranden saveh	Middle East	Iran	Davis, CA, USA
88	Toryu-shibori	East Asia	Japan	Davis, CA, USA
89	Vishnevyi	Central Asia	Turkmenistan	Davis, CA, USA
90	Vkusnyi	Central Asia	Turkmenistan	Davis, CA, USA
91	White Flower	Central Asia	Turkmenistan	Davis, CA, USA
92	Wonderful-1	Unknown	Unknown	Davis, CA, USA
93	Wonderful-2	North America	United States	Davis, CA, USA
94	Zubejda	Central Asia	former Soviet Union	Davis, CA, USA

**Table 2:** SSR markers used in this study. Locus code, melting temperature, fragment length range, number of alleles, PIC, observed (Ho) and nonbiased expected (He [21]) heterozygosity values are shown. Primer sequences can be checked at [15].

Locus	Melting Temp. (°C)	Fragment Length Range (bp)	No. Alleles	PIC	Ho	He
PGCT015	60	164–228	7	0.627	0.511	0.630
PGCT016	57	196–204	3	0.607	0.538	0.611
PGCT022	55	238–243	4	0.178	0.172	0.179
PGCT028	57	228–240	4	0.407	0.352	0.409
PGCT032	59	123–129	3	0.434	0.430	0.437
PGCT038	57	265–284	5	0.460	0.380	0.460
PGCT057	56	178–182	3	0.516	0.473	0.519
PGCT066	59	124–131	3	0.481	0.355	0.483
PGCT083	59	131–143	5	0.494	0.484	0.497
PGCT087	58	253–263	5	0.671	0.457	0.674
PGCT088A	59	148–169	5	0.604	0.451	0.607
PGCT088B	59	246–269	5	0.596	0.348	0.600
PGCT089	59	134–154	7	0.603	0.511	0.606
PGCT091	56	203–223	9	0.638	0.462	0.642
PGCT093A	58	231–254	7	0.658	0.554	0.662
PGCT093B	60	182–208	7	0.599	0.511	0.602
PGCT098	60	114–146	4	0.365	0.319	0.367
PGCT110	52	104–188	13	0.618	0.500	0.622
PGCT111	58	206–248	6	0.653	0.495	0.656

**Table 3:** Putative redundant pomegranate accessions according to the genetic profile of the 19 SSRs used in this study.

Group	Accession Number (Region)
G1	6 (EE), 34 (NA), 63 (EE)
G2	12 (TR), 20 (NA), 47 (TR), 74 (CA)
G3	21 (NA), 51 (CA)
G4	23 (ME), 32 (ME)
G5	58 (TR), 75 (EE)
G6	14 (SE), 17(SE)

EE: Eastern Europe; NA: North America; TR: Transcaucasia; CA: Central Asia; ME: Middle East; SE: Southern Europe.

**Table 4:** Genetic diversity found in the accessions grouped by their geographic region of origin.

Group	No. Accs	MLG	eMLG	A	Exclusive Alleles	Hobs	Hexp	Corrected Simpson’s Index
North America	15	15	10.00	3.74	5	0.370 (0.110)	0.548	1.000
Southern Europe	13	12	9.42	2.47	2	0.531 (0.258)	0.440	0.987
Eastern Europe	8	7	7.00	2.63	0	0.428 (0.284)	0.410	0.964
Transcaucasia	5	4	4.00	2.63	0	0.558 (0.356)	0.483	0.900
Middle East	7	6	6.00	2.42	0	0.471 (0.245)	0.479	0.952
Central Asia	35	35	10.00	4.32	11	0.414 (0.116)	0.486	1.000
South Asia	1	1	1.00	1.32	5	0.316 (0.478)	0.316	NA
East Asia	5	5	5.00	2.84	6	0.463 (0.291)	0.488	1.000
Unknown	5	5	5.00	2.84	0	0.421 (0.266)	0.473	1.000
Total	94	85	9.87	5.47	29		0.540	0.997

MLG: number of multilocus genotypes found in the specified population; eMLG: expected number of MLG at the lowest common sample size; A: mean number of alleles per locus; Hobs: observed heterozygosity; Hexp: unbiased estimated heterozygosity (Nei’s gene diversity).

## Data Availability

Not applicable.

## Supplementary Materials

The following are available online at https://www.mdpi.com/article/10.3390/plants11091257/s1, Figure S1: First Factorial Correspondence Analysis (FCAs) of the 85 accessions analyzed using 19 SSRs. Colors represent the putative region of origin of the genotype; Figure S2: Bayesian-based population assignment allowing admixture carried out using the Structure software. (A) K = 2 based on delta K method, (B) K = 10 based on the maximum likelihood of K, (C) Delta K (ΔK) graph obtained by Structure Harvester, (D) mean likelihood L(K) and variance per K value obtained by Structure Harvester; Table S1: Description of the morphological and pomological data of the analyzed accessions; Table S2: Genotypic profile of the 94 accessions analyzed with 19 SSRs; Table S3: Coordinates of each accession in the first 3 dimensions of each of the FCAs conducted.
