# Supplementary figures and images for: Characterization of the Spanish Pomegranate Germplasm Collection Maintained at the Agricultural Experiment Station of Elche to Identify Promising Breeding Materials
Source: Plants (Basel). 2022 May 6;11(9):1257. doi: 10.3390/plants11091257 (PMC9101082; doi:10.3390/plants11091257)

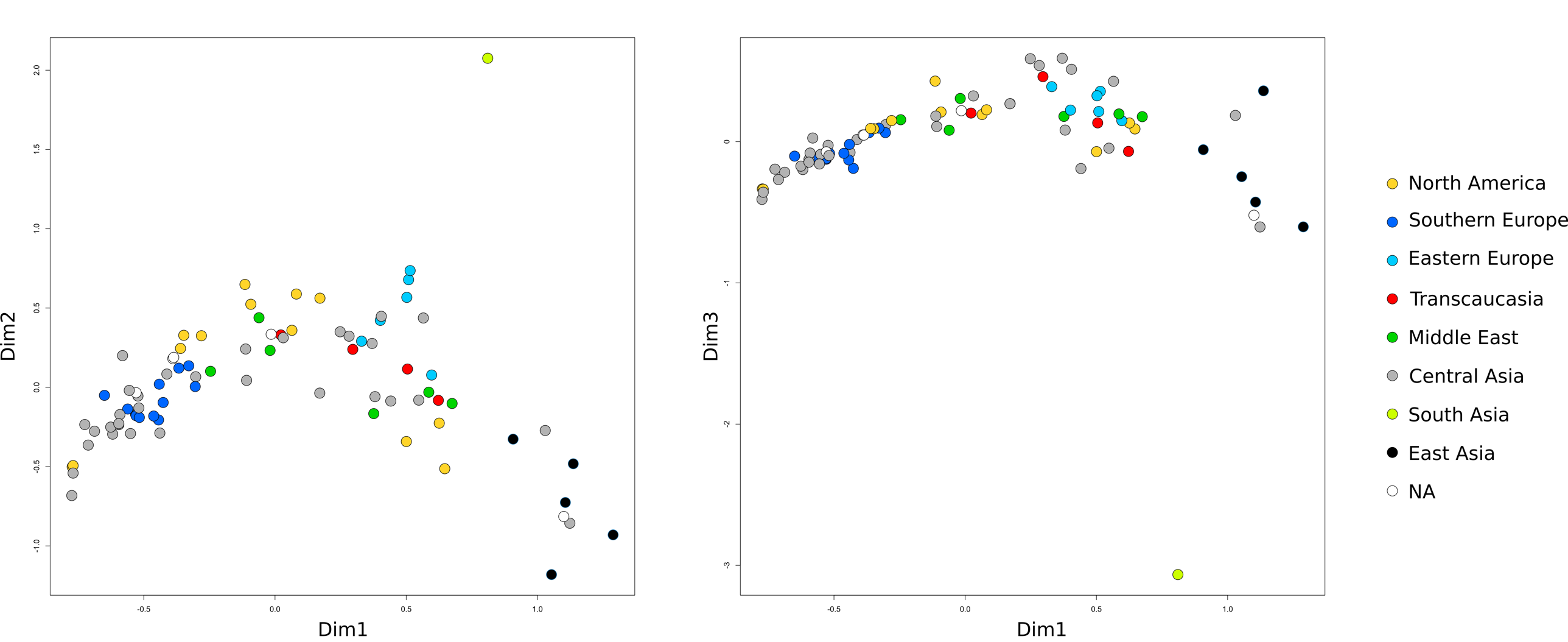

Supplement: Supplementary file 1 [file plants-11-01257-s001.zip › plants-1701919-supplementary/Figure_S1_AFC1.png]

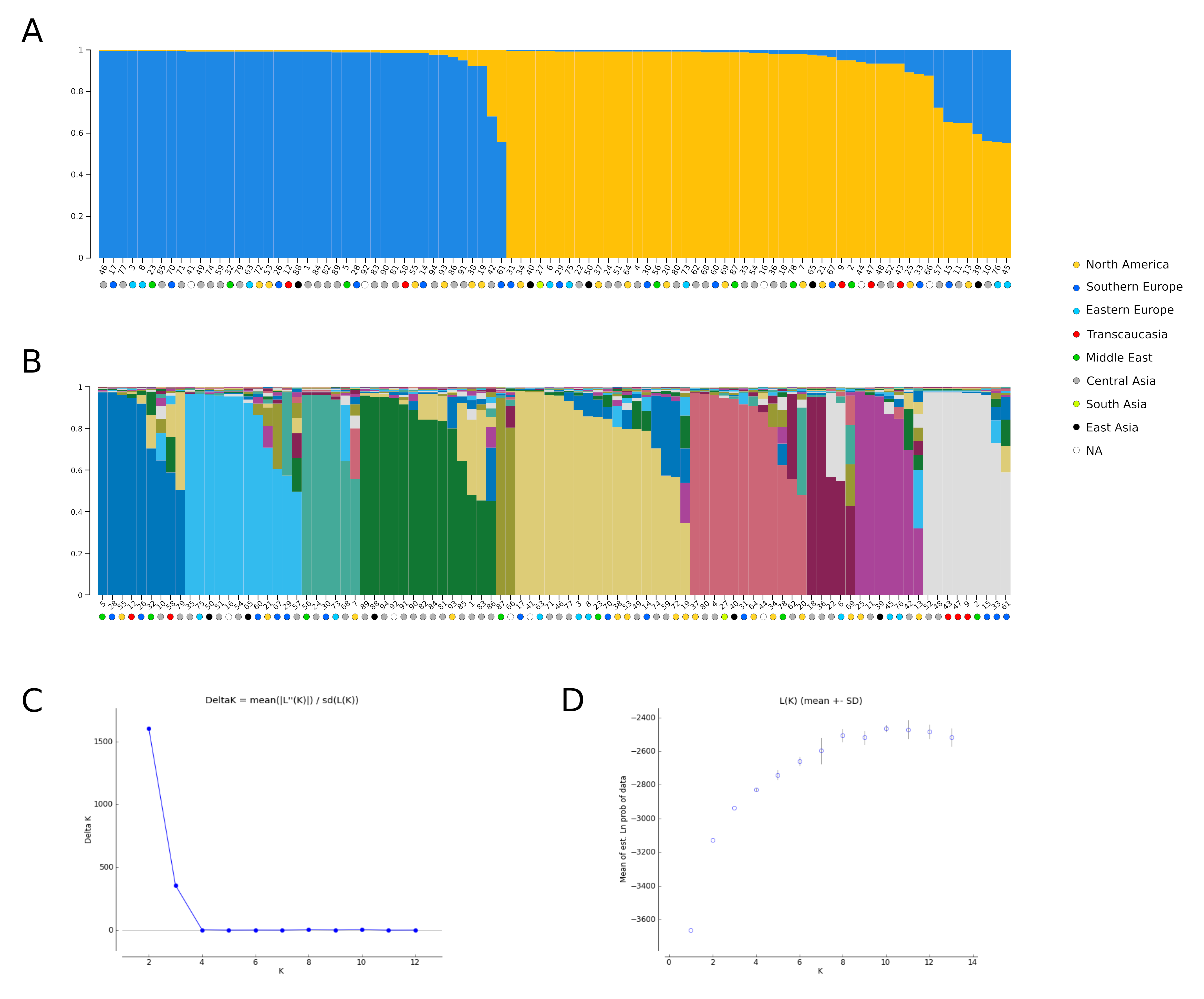

Supplement: Supplementary file 1 [file plants-11-01257-s001.zip › plants-1701919-supplementary/Figure_S2.png]
